# Supplementary material for: Membrane remodeling by FAM92A1 during brain development regulates neuronal morphology, synaptic function, and cognition
Source: Nat Commun. 2024 Jul 23;15:6209. doi: 10.1038/s41467-024-50565-w (PMC11266426; doi:10.1038/s41467-024-50565-w)
Supplement: Supplementary file 1 — Supplementary Information [file 41467_2024_50565_MOESM1_ESM.pdf]

Supplementary information

Membrane remodeling by FAM92A1 during brain development regulates neuronal morphology, synaptic function, and cognition

Liang Wang[1,2#], Ziyun Yang[1#], Satoshi Fudo[3], Xavier Prasanna[4], Ziyi Yan[2], Helena Vihinen[5], Yaxing Chen[1], Yue Zhao[1], Xiumei He[1,6,7], Qian Bu[1], Hongchun Li[1], Ying Zhao[1], Linhong Jiang[1], Feng Qin[1], Yanping Dai[1], Ni Zhang[8], Meng Qin[1], Weihong Kuang[8], Yinglan Zhao[1], Eija Jokitalo[5], Ilpo Vattulainen[4], Tommi Kajander[3], Hongxia Zhao[2,6,7*], Xiaobo Cen[1,*]

[1] Mental Health Center and National Chengdu Center for Safety Evaluation of Drugs, State Key Laboratory of Biotherapy, West China Hospital of Sichuan University, Chengdu 610041, China.

[2] Faculty of Biological and Environmental Sciences, University of Helsinki, 00014 Helsinki, Finland.

[3] Helsinki Institute of Life Science - Institute of Biotechnology, University of Helsinki, Finland.

[4] Department of Physics, University of Helsinki, Helsinki, Finland.

[5] Helsinki Institute of Life Science (HiLIFE) - Institute of Biotechnology, University of Helsinki, Helsinki, Finland.

[6] School of Life Sciences, Guangxi Normal University, Guilin, China.

[7] Guangxi Universities Key Laboratory of Stem Cell and Biopharmaceutical Technology, Guangxi Normal University, Guilin 541004, China

[8] Mental Health Center, West China Hospital, Sichuan University, Chengdu, Sichuan 610041, China.

[*] Corresponding authors:
E-mail: xbcen@scu.edu.cn (X.C.); hongxia.zhao@helsinki.fi (H.Z.)
[#] These authors contributed equally to this work.

This PDF file includes:

Supplementary Figures 1 to 8

Supplementary Tables 1 to 4

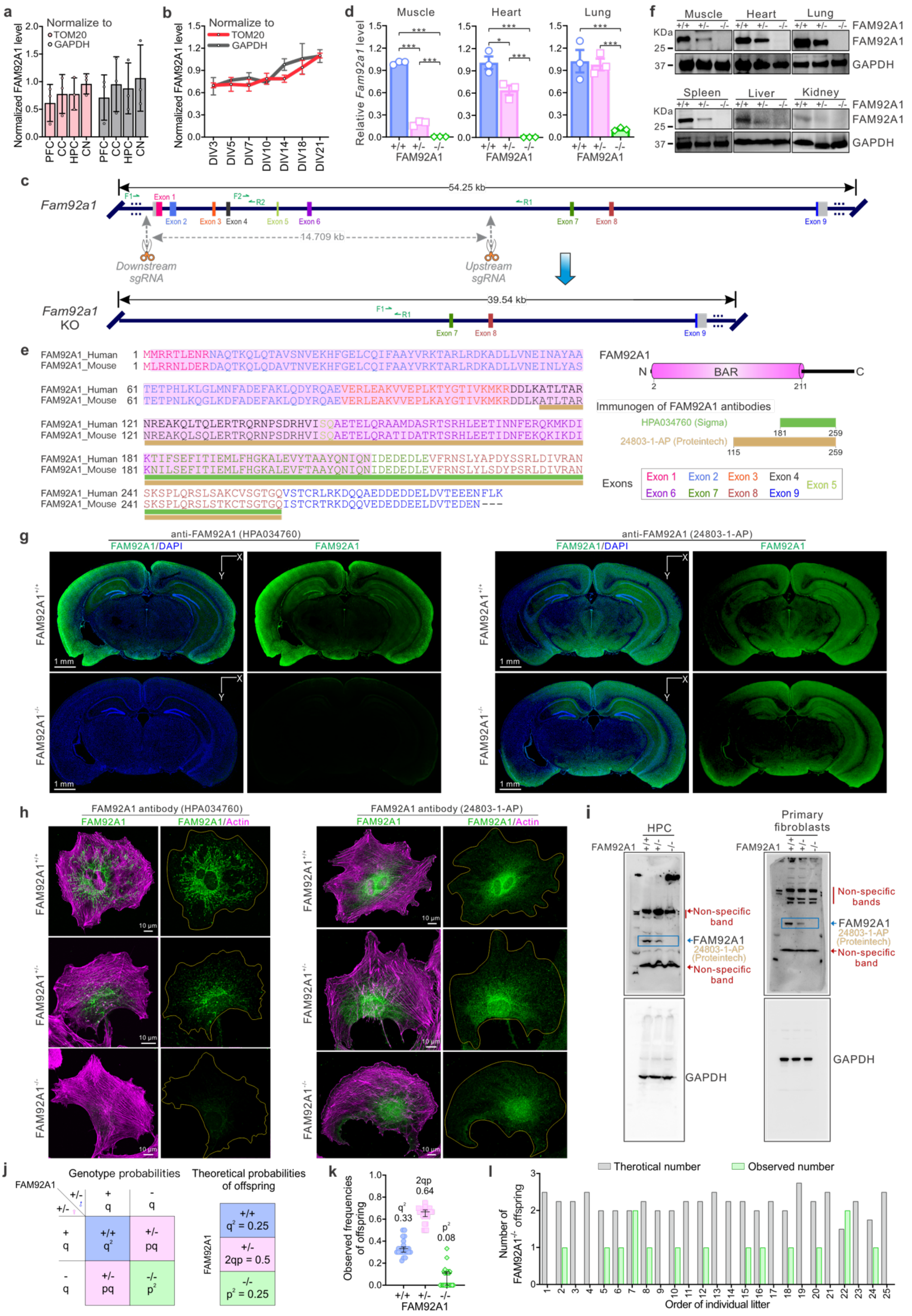

Supplementary Fig. 1 The strategy of editing mouse *Fam92a1* allele and the identification of FAM92A1 expression level.

a, b Quantification of FAM92A1 levels in various brain regions of mice (**a**) and primary hippocampal neurons at different culture days (**b**). Data represent mean ± SEM of three biologically independent experiments. **c** Schematic diagram illustrating the construction strategy of FAM92A1 knockout mouse. The targeted positions of the two sgRNAs and the primers used for genotyping were separately marked on the schematic diagram. **d** RT-qPCR analysis of FAM92A1 expression within the muscle, heart, and lung tissues. Data present mean ± SEM of three technical replicates over one independent experiment (one-way ANOVA). **e** Sequence alignment of FAM92A1 between human and mouse using the Clustal Omega server. Sequences encoded by corresponding exons are presented in different text colors, FAM92A1 BAR domain is marked with magenta, and the immunogen sequences used for the generation of two anti-FAM92A1 antibodies are separately shown below the aligned sequences. **f** Western blot analysis of FAM92A1 expression within the indicated tissues. **g, h** Representative images across three biologically independent experiments showing FAM92A1 expression in coronal section of brain slices (**g**) and primary fibroblasts (**h**) from FAM92A1-deficient mice and wild-type littermates. Scale bars, 1 mm (**g**) or 10 μm (**h**). **i** Western blot analysis of protein signals recognized by the FAM92A1 antibody (24803-1-AP) in the hippocampus and primary fibroblasts. **j** Mendelian model of the progenies of a single breeding of two FAM92A1 heterozygous mice. + and − represent *Fam92a1* alleles occurring with probabilities q and p, respectively. **k** Dot plot showing the observed frequencies of three FAM92A1 genotypes across 25 litters. **l** Bar graph showing the theoretical and observed numbers of FAM92A1$^{-/-}$ offspring in each litter. *$p < 0.05$ and ***$p < 0.001$. Source data and exact p values are provided as a Source Data file.

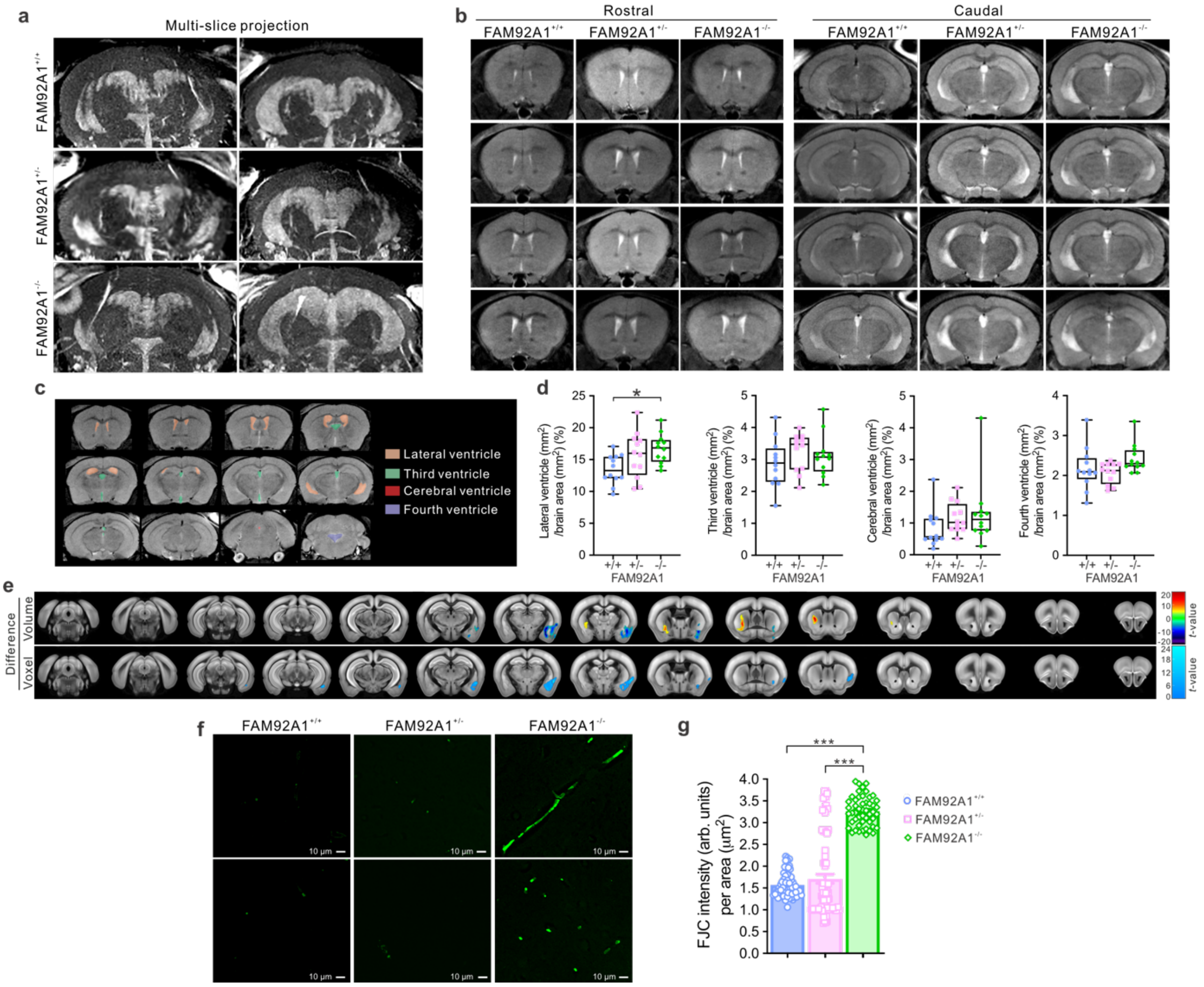

Supplementary Fig. 2 FAM92A1 depletion causes abnormal brain structure and degeneration of hippocampal neuron.

a, b Representative MRI images of multi-slice maximum intensity projections (**a**) and single rostral and caudal slices (**b**). **c** Representative coronal view of MRI images with marked lateral ventricle (orange), third ventricle (light green), cerebral ventricle (red), and fourth ventricle (light purple). **d** Quantification showing the area of indicated ventricles for each FAM92A1 genotyping group. Data are presented minimum to maximum values (whiskers) and the 25th to 75th percentiles (box), with the mean value within the box; n = 12 mice per group, one-way ANOVA. **e** Visualization of significant volume differences (top) and voxel differences (bottom) in coronal planes between the FAM92A1$^{+/+}$ and FAM92A1$^{+/-}$ groups. Results are presented as *t*-maps that were cluster-corrected for multiple comparisons using voxel $p < 0.01$ and cluster $p < 0.05$, family-wise error (FWE) correction < 0.05. n = 3 mice per group. The color bar represents the *t*-value of unpaired two-tailed Student's *t* test. For the DBM-based volume difference, the color bar from the green to red and blue separately represent the increased and decreased volume in FAM92A1 heterozygous mice; for the VBM-based voxel difference, the color indicates the decreased voxel density in FAM92A1 heterozygous mice. **f, g**

Representative images (**f**) and quantification (**g**) of FJC staining of mouse brain. Scale bar, 10 μm. Data present as mean ± SEM; n = 50–78 measurements from two mouse brains per group, one-way ANOVA. **p* < 0.05 and ****p* < 0.001. Source data and exact *p* values are provided as a Source Data file.

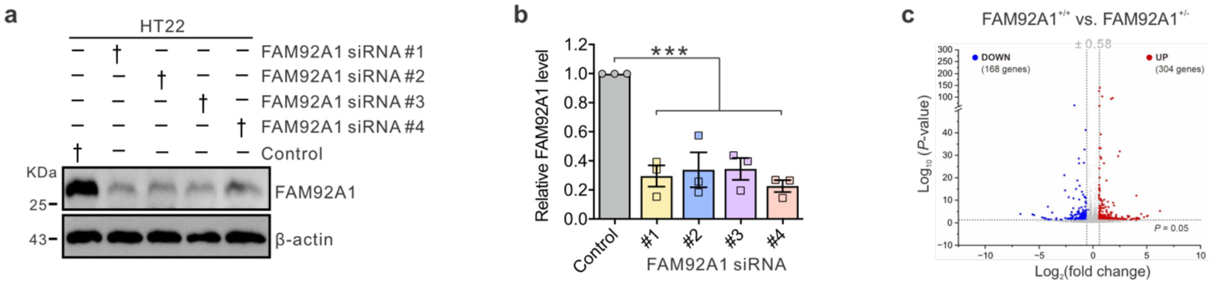

Supplementary Fig. 3 FAM92A1 is efficiently silenced and the hippocampal DEGs after heterozygous loss of FAM92A1.

a, b Western blot analysis (**a**) and quantification (**b**) of FAM92A1 expression in HT22 cells after treated with control and different FAM92A1 siRNAs for 72 h. Data represent mean ± SEM of three biologically independent experiments; one-way ANOVA. **c** Volcano plot showing the differentially expressed genes (DEGs) (fold change > 1.5 and *p* < 0.05) of RNA-sequencing upon heterozygous loss of FAM92A1. ****p* < 0.001. Statistical test was based on a two-sided *t* test with *P*-value adjustment for multiple comparisons (Benjamini–Hochberg method) by DESeq2 (n = 3 biologically independent samples per group). Source data and exact *p* values are provided as a Source Data file.

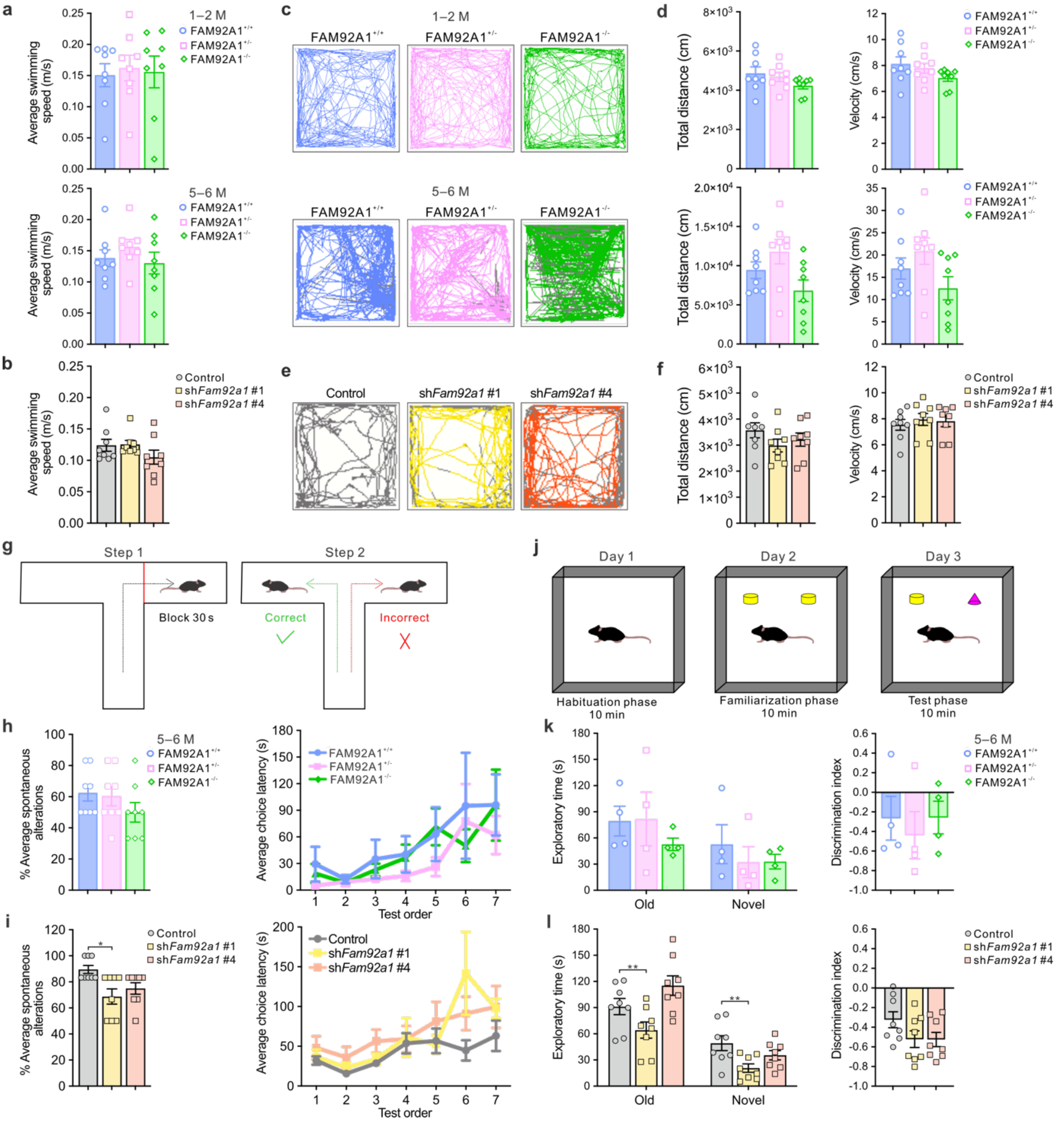

Supplementary Fig. 4 FAM92A1 depletion causes age-associated memory decline and cognitive deficits.

a, b Bar graphs showing the average swimming speed of FAM92A1 knockout (**a**) and knockdown mice (**b**) during the probe trial. Data represent mean ± SEM; n = 8 mice per group; one-way ANOVA. **c-f** Representative trajectories (**c, e**) and quantification analysis (**d, f**) of total distance (cm) and velocity (cm/s) traveled by FAM92A1 knockout (**c, d**) and knockdown mice (**e, f**) within 15 min recording time in the apparatus of the open field test. Data represent mean ± SEM; n = 8 mice per group; one-way ANOVA. **g** Experimental diagram for the T-maze test. **h, i** Bar graphs showing the percentage of alteration of FAM92A1 knockout (**h**) and knockdown mice (**i**) within the first 6 trials (left) (one-way ANOVA). Line graphs on the right showing the choice latency (sec) of FAM92A1 knockout (**h**) and knockdown mice (**i**) (two-

way ANOVA). Data represent mean ± SEM; n = 8 mice per group. **j** Experimental diagram for the novel object recognition test. **k, l** Bar graphs showing the exploratory time for old and novel objects by FAM92A1 knockout (**k**) and knockdown mice (**l**) within 15 min recording time (left, two-way ANOVA) and the quantified discrimination index (right, one-way ANOVA). Data represent mean ± SEM; n = 4 mice per group for panel **k** and n= 8 mice per group for panel. *$p < 0.05$. Source data and exact p values are provided as a Source Data file.

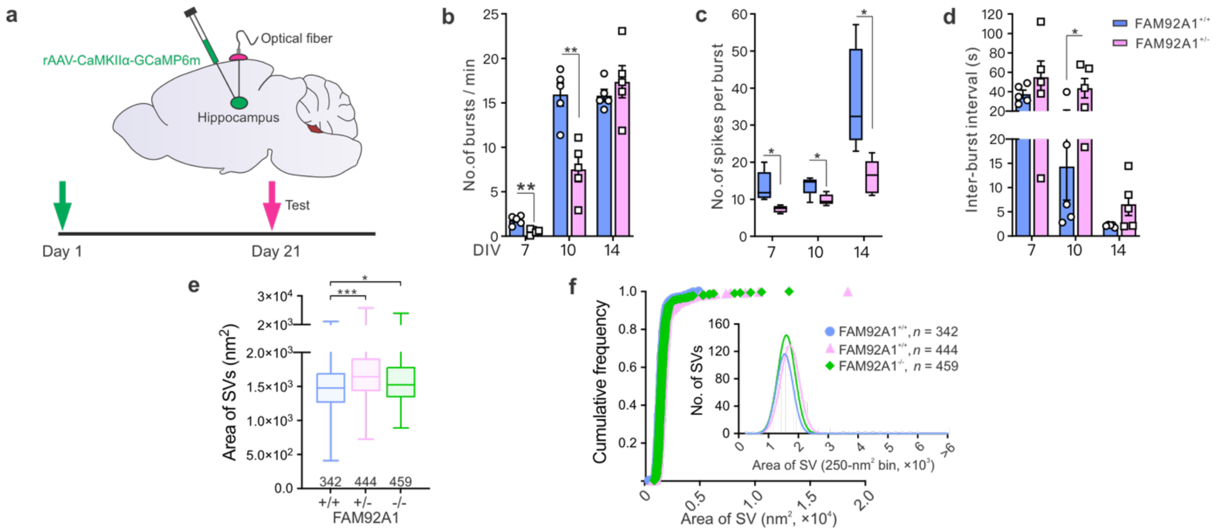

Supplementary Fig. 5 FAM92A1 deficiency impairs neuronal function and synaptic structure. **a** Schematic diagram showing injection of rAAV9- CaMKIIα-GCaMP6m, implantation of optical fiber, and the experimental timeline. **b** Bar graph showing the number of bursts per minute. Data represent mean ± SEM; n = 5 MEA wells per group; multiple unpaired *t* tests. **c** Box plot showing the number of spikes per burst. Data presented as minimum to maximum values (whiskers) and the 25th to 75th percentiles (box), with the mean value within the box; n = 5 MEA wells per group; multiple unpaired *t* tests. **d** Bar graph showing the inter-burst interval (sec). Data represent mean ± SEM; n = 5 MEA wells per group; multiple unpaired *t* tests. **e** Box plot showing the area of SVs. Data presented as minimum to maximum values (whiskers) and the 25th to 75th percentiles (box), with the mean value within the box. one-way ANOVA. **f** Cumulative frequency plot showing the change in the SV area. The inset of the Gaussian curve showing the enlarged SVs in the FAM92A1-deficient mice. For **e** and **f**, SVs (n = 342 for FAM92A1$^{+/+}$ group, n = 444 for FAM92A1$^{+/-}$ group, and n = 459 for FAM92A1$^{-/-}$ group) from 15 synapses per group. *$p < 0.05$ and ***$p < 0.001$. Source data and exact p values are provided as a Source Data file.

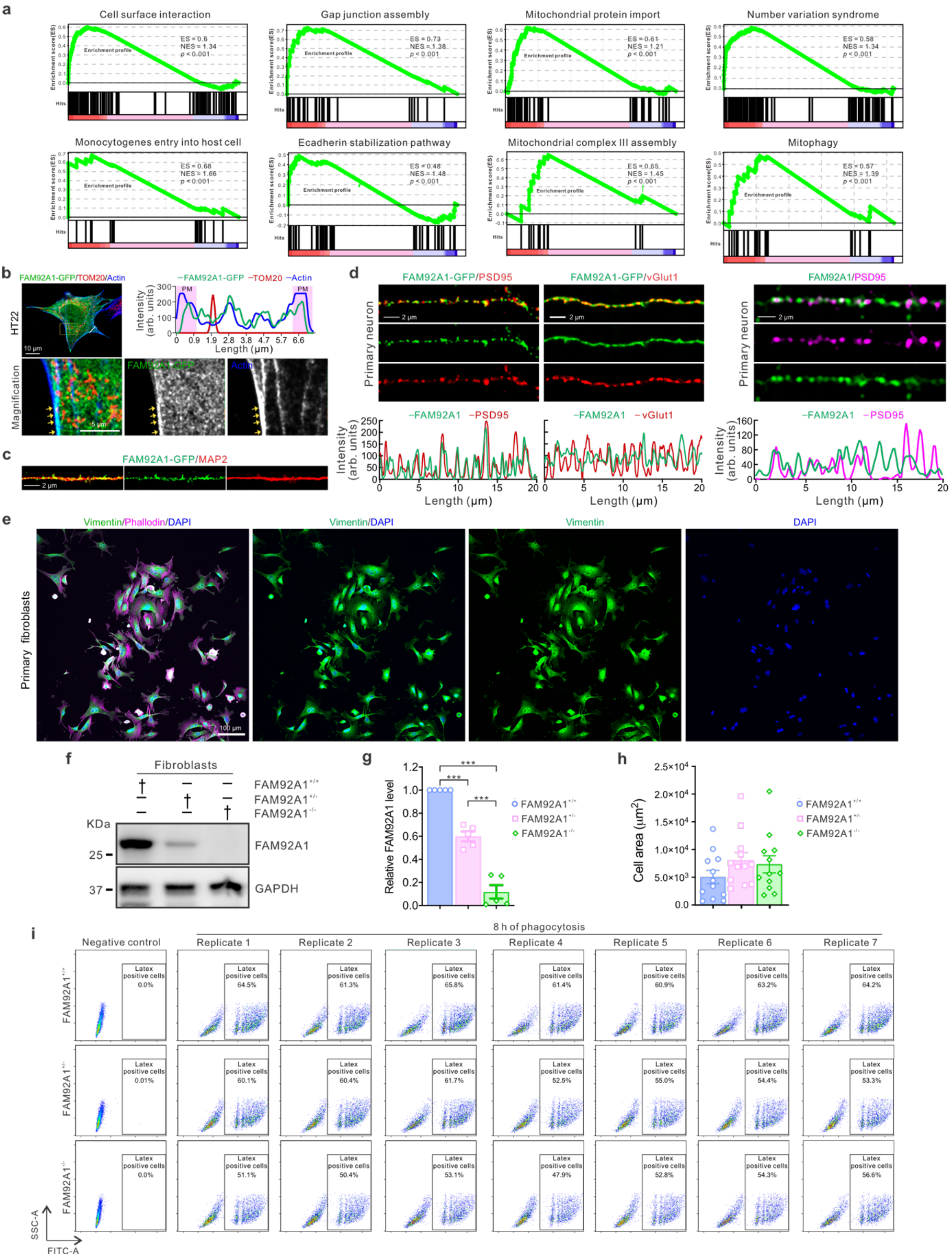

Supplementary Fig. 6 FAM92A1 deficiency results in the enrichment of membrane-associated pathways and it localizes to synapses engaging in endocytosis.

a GSEA enrichment score curves of differentially expressed genes following the depletion of FAM92A1. ES, enrichment score; NES, normalized enrichment score. **b** Representative images showing the distribution of FAM92A1-EGFP after overexpressed in HT22 cells for 48

h. Mitochondria and actin meshwork near the plasma membrane were visualized by TOM20 and phalloidin, respectively. Line graph showing the fluorescence intensity profiles of the indicated white line. Scale bar, 10 μm (upper) and 5 μm (lower). **c** Representative images showing the distribution of overexpressed FAM92A1-GFP in primary hippocampal neurons. Scale bar, 2 μm. **d** Representative images showing the co-localization of overexpressed FAM92A1-GFP and endogenous FAM92A1 with mitochondrial and synaptic proteins. Scale bar, 2 μm. Line graph below showing the fluorescence intensity profiles of the indicated proteins. **e** Representative images showing the purity of the primary fibroblasts using the antibody against vimentin. Scale bar, 100 μm. For **b–e**, representative images are from at least three biologically independent experiments. **f, g** Western blot (**e**) and quantification (**g**) of FAM92A1 expression in the primary fibroblasts. Data represent mean ± SEM of five biologically independent experiments; one-way ANOVA. **h** Bar graphs showing the area (μm^2) of fibroblasts. Data represent mean ± SEM; n = 12 fibroblasts per group from two biologically independent experiments; one-way ANOVA. **i** Representative images of the phagocytic capacity of fibroblasts using the fluorescence-labeled latex beads. $^{***}p < 0.001$. Source data and exact *p* values are provided as a Source Data file.

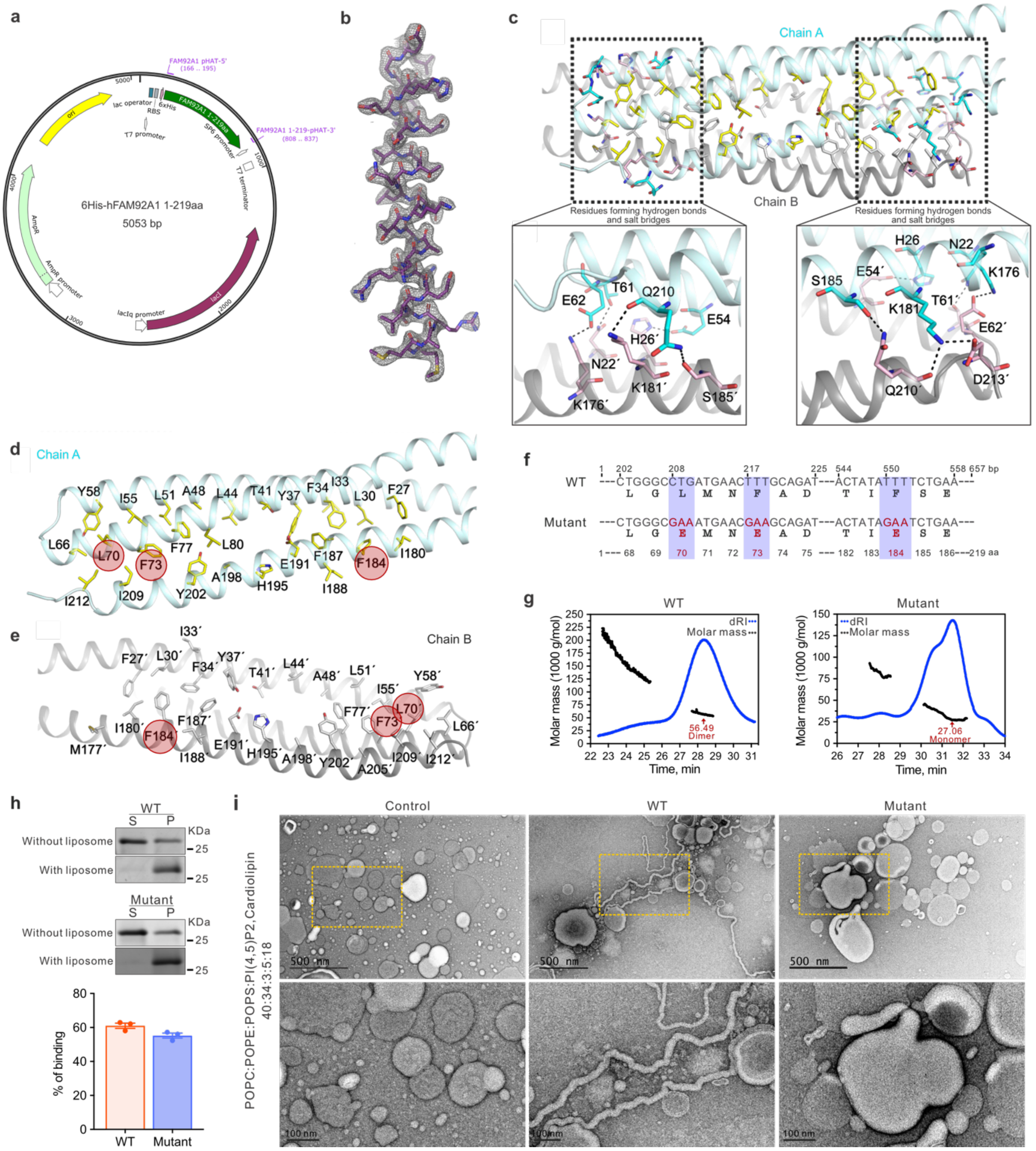

Supplementary Fig. 7 FAM92A1 dimer interface and mutation the residues at the dimer interface disrupt the formation of dimer.

a Plasmid map used for the expression of FAM92A1 1-219aa. Primers for sequencing are labeled in purple text. **b** The 2*F*o-*F*c electron density map of FAM92A1 residues 1-29 contoured at 1.0 σ level. **c** Overall view of the FAM92A1 dimer interface with all the residues at the interface shown as sticks. Residues forming hydrogen bonds and/or salt bridges are magnified and shown in cyan and pink for chains A and B, respectively. Backbone cartoon presentations of chains A and B are in pale cyan and gray, respectively. Hydrogen bonds and salt bridges are indicated by black dashed lines. **d** Buried residues at the dimer interface only

for chain A. **e** Buried residues at the dimer interface only for chain B. For **d** and **e**, the same color annotation and orientation were applied as **c** with the residue numbering. For **c**–**e**, the prime (′) after the residue numbers denotes chain B, whereas no prime denotes chain A. **f** Schematic diagram showing the sites of mutations. **g** SEC-MALS profile showing the measured molar mass of WT and mutant. The major peak in the chromatogram corresponds to a dimeric species for the wild-type protein and a monomeric species for the mutant. dRI: differential refractive index. **h** (Up) Gel images showing the co-sedimentation assay for the interaction of FAM92A1 BAR domain WT and mutant with MIM liposomes, respectively. (Down) Bar graph showing the quantified binding ability of WT and mutant with the liposomes. Data represent mean ± SEM of three biologically independent experiments (unpaired two-tailed Student's *t* test). **i** Electron micrographs showing the membrane tubulation of unilamellar vesicles. Source data and exact *p* values are provided as a Source Data file.

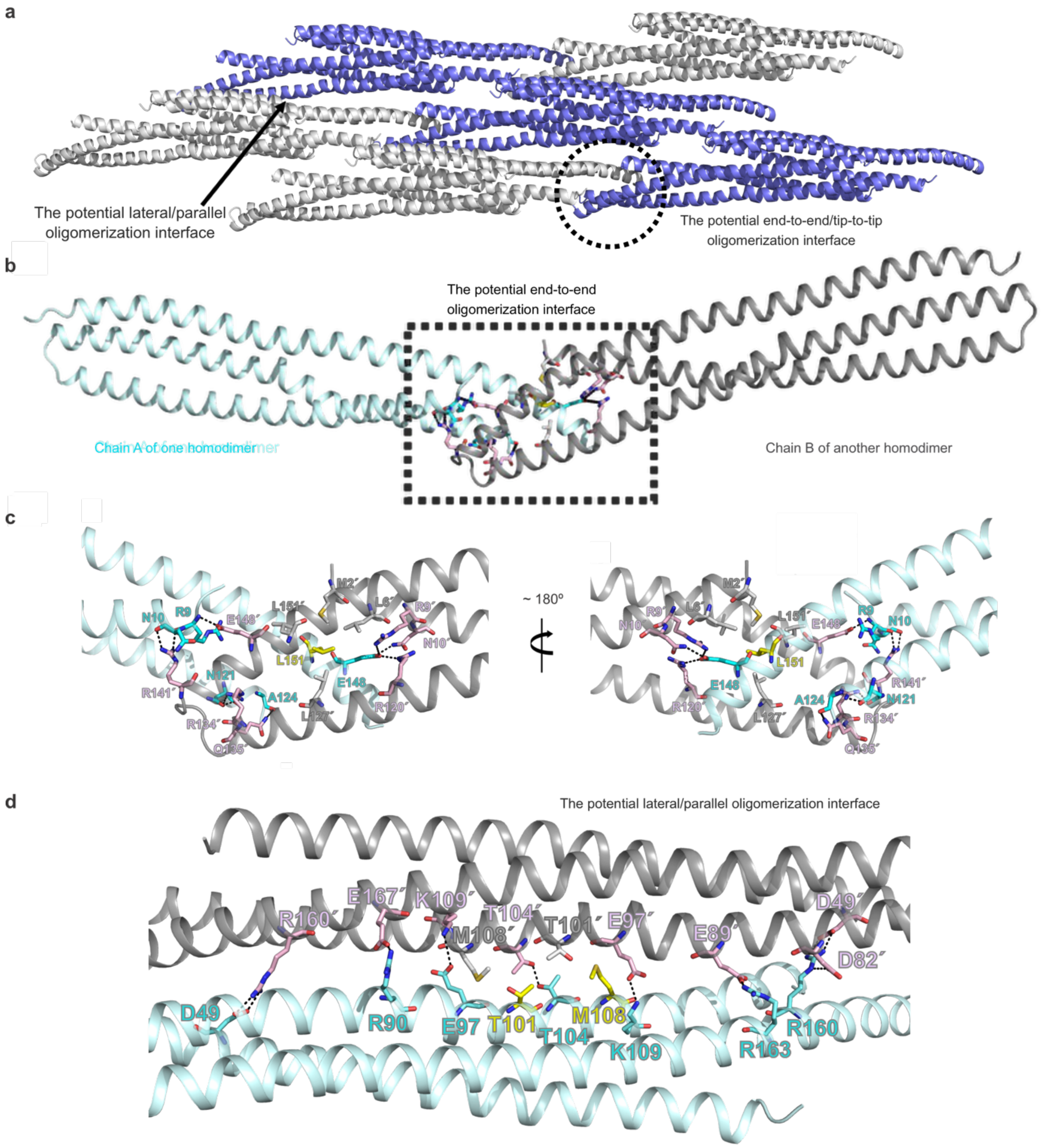

Supplementary Fig. 8. FAM92A1 oligomer interface.

a Overview of the crystal lattice formed by the FAM92A1 BAR domain dimers (adjacent dimers in light grey and blue) and through the end-to-end oligomerization interfaces (circled) and other lateral interfaces (arrow), providing a potential model for oligomeric assembly of FAM92A1 BAR domain in the membrane environment, end-to-end contacts in BAR domains have been characterized as feature associated to their oligomerization. Additional lateral assembly could be envisioned to induce larger aggregates on the membrane surface (though the PISA-server ranked the lateral interfaces as not significant in terms of the score and ΔG for contact formation). **b** Overall view of two FAM92A1 monomers from different adjacent

asymmetric units forming the potential oligomer interface (indicated by the dotted square box) with all the residues at the interface shown as sticks. Residues forming hydrogen bonds and/or salt bridges are shown in cyan and pink for chains A and B, respectively, whereas those that are buried at the interface are shown in yellow and gray for chains A and B, respectively. Backbone cartoons of chains A and B are in pale cyan and gray, respectively. **c** Magnified view of the potential tip-to-tip oligomer interface with the residue numbering. The same color annotation was applied as **b**. The prime (′) after the residue numbers denotes chain B, whereas no prime denotes chain A. Hydrogen bonds and salt bridges are indicated by black dashed lines. The views of the left panel and right panel are related by a 180-degree rotation. **d** Potential lateral oligomerization interface formed by packing of the molecules in the crystal. Same annotation was used as in **c**.

Supplementary Table 1. Diffraction data collection and structure refinement.

**Data collection**	
Beam line	I24 Diamond
Wavelength (Å)	0.89998
Space group	*P*1
Cell dimensions	
a, *b*, *c* (Å)	49.04, 54.92, 57.51
α, β, γ (°)	88.32, 67.40, 63.86
No. reflections	106116
Unique reflections	30902
Resolution (Å)	30–2.03 (2.08–2.03)
R_{merge}	0.71 (0.73)
$I / \sigma I$*	11.0 (1.7)
$CC_{1/2}$ (%) *	99.8 (73.1)
Redundancy	3.4 (3.4)
Completeness (%) *	97.4 (94.7)
**Refinement**	
Resolution (Å)	30–2.03
No. reflections	30855
R_{work} / R_{free}	0.18 / 0.23
No. atoms	
Protein	3225
Water	156
B-factors	
Protein	45.9
Water	45.8
R.m.s. deviations	
Bond lengths (Å)	0.008
Bond angles (°)	1.4826

Ramachandran plot (favored/allowed) (%)	98.75 / 1.25

Note: * High-resolution shell value in parentheses (2.08-2.03)

Supplementary Table 2. The lipid compositions of model bilayers.

Name of lipid	% membrane composition
MIM bilayer	
POPC	40%
POPE	34%
POPS	3%
$PI(4,5)P_2$	5%
Cardiolipin	18%
PIP_2 bilayer	
POPC	48%
POPE	34%
$PI(4,5)P_2$	18%
Cardiolipin bilayer	
POPC	48%
POPE	34%
Cardiolipin	18%

Note: POPC, 1-palmitoyl-2-oleoyl-sn-glycero-3-phosphocholine; POPE, 1-palmitoyl-2-oleoyl-sn-glycero-3-phosphoethanolamine; POPS, 1-palmitoyl-2-oleoyl-sn-glycero-3-phospho-L-serine; $PI(4,5)P_2$, 1-palmitoyl-2-oleoyl-sn-glycero-3-phosphatidylinositol 4,5-bisphosphate; Cardiolipin, tetralinoleoyl cardiolipin.

Supplementary Table 3. Details of simulation.

System	No. of simulations	Simulation time (μs)
FAM92A1 monomer + PIP2 bilayer	10	0.5
FAM92A1 monomer + Cardiolipin bilayer	10	0.5
PIP_2 bilayer	3	1
Cardiolipin bilayer	3	1
MIM bilayer	3	1
FAM92A1 dimer + PIP2 bilayer	3	1
FAM92A1 dimer + Cardiolipin bilayer	3	1
FAM92A1 dimer + MIM bilayer	3	1

Supplementary Table 4. Primers used for RT-qPCR assay and generating mutant construct.

Name	Sequence (5'-3')
Fam92a1-F	5'- CTGGCAGTTTTCCGTACATAAG -3'
Fam92a1-R	5'- GATGCTCAAACAAAACAACTGC -3'
Slc32a1-F	5'- CTTCGTCATCGGCGGCATCTG -3'
S lc32a1-R	5'- TAGTCCTCTGCGTTGGTTCGGTAG -3'
Syn1-F	5'- CATTCTGGGATGGGCAAGGTCAAG -3'
Syn1-R	5'- GGCTCAGCAGTGGCATATGTCTTAG -3'
Slc17a7-F	5'- TACTGGAGAAGCGGCAGGAAGG -3'
S lc17a7-R	5'- GATGGCGATGATGTAGCGACGAG -3'

Gad2-F	5'- CAGTGTGGACGCCATGTGGATG -3'
Gad2-R	5'- AATGTGTGCCTCAAACCCAGTAGTC -3'
Synj1-F	5'- GGTGGGTGTCTGCCTGTTTGTC -3'
Synj1-R	5'- TGGTGTGGAAGAGCATCCGAATTG -3'
Syt1-F	5'- ATCTGCTTCTCCCTCCGCTACG -3'
Syt1-R	5'- GCCACCCACATCCATCTTCTTCAG -3'
Rab3a-F	5'- TCCGCCACAGACTCTCGCTATG -3'
Rab3a -R	5'- CCCACGCTGCTGTTCCCAATG -3'
Syp-F	5'- GATGCCTATGTGCCGCCAGAC -3'
Syp-R	5'- GCCTGTCTCCTTGAACACGAACC-3'
Vamp2-F	5'- CCTGCACCTCCTCCAAACCTTAC -3'
Vamp2-R	5'- GGTCATCCAGCTCCGACAACTTC-3'
Snap91-F	5'- TACCACCACTGCCACCACCTC -3'
Snap91-R	5'- GTGCCAAACAGGTCTATGCTAGGAG-3'
Ap2a2-F	5'- AGGGCGGTTAGTGGAGTGTCTG -3'
Ap2a2-R	5'- ATAGCGTTCTTGGCGTTGGAGTG -3'
Hspa8-F	5'- ATTGCAGAAGCGTACCTCGGAAAG -3'
Hspa8-R	5'- GCAGCAGCAGTTGGTTCATTGATG-3'
Clta-F	5'- CGGATGCTGTTGACGGAGTGATG -3'
Clta -R	5'- TCTGAAATGGCTGCGTAACTGTCTG -3'
Cltc-F	5'- GGCGAGTATCAGGCAGCAGTTG -3'
Cltc-R	5'- CCCATCTACACAGGCAAAGCAGAC -3'
Endoa2-F	5'- AGAATCCAGCAACAACAGGAAGGC -3'
Endoa2-R	5'- CCATTAGCCAGTCGGAGTCCATTC -3'
Snx9-F	5'- CAGAGCTTAGCGGCAGTGTTCAG -3'
Snx9-R	5'- AGGCTGGCGATCTCCTCATACG-3'
Pacsin1-F	5'- ACACCACTGCCAAGAAGGAGAAAC -3'
Pacsin1-R	5'- GCCTCGGTCATAGCTGCTAACAC -3'
Dnm2-F	5'- AGGACCAGGCAGAGAATGAGGATG -3'
Dnm2-R	5'- ACGGATGGACTTGTTGATGATGGC -3'
Mutant (L70E)-F	5'- GCTGGGCGAGATGAACTTTGCAGATGAGTTTGCC -3'
Mutant (L70E)-R	5'- GTTCATCTCGCCCAGCTTTAAATGCGGGGTCTC -3'
Mutant (F73E)-F	5'- GATGAACGAGGCAGATGAGTTTGCCAAACTTCAGG -3'
Mutant (F73E)-R	5'- CATCTGCCTCGTTCATCAGGCCCAGCTTTAAATG -3'
Mutant (F184E)-F	5'- GACTATAGAGTCTGAATTTATCACAATCGAAATG -3'
Mutant (F184E)-R	5'- CAGACTCTATAGTCTTTATATCCTTCATTTTCTG -3'
