# Supplementary material for: Membrane remodeling by FAM92A1 during brain development regulates neuronal morphology, synaptic function, and cognition
Source: Nat Commun. 2024 Jul 23;15:6209. doi: 10.1038/s41467-024-50565-w (PMC11266426; doi:10.1038/s41467-024-50565-w)
Supplement: Supplementary file 3 — Description of Additional Supplementary Information [file 41467_2024_50565_MOESM3_ESM.docx]

**Legends for each Supplementary Movie file**

**Supplementary Movie 1. FM1-43 endocytic process by fibroblasts from FAM92A1^+/+^ mice.**

Time-lapse movie of FM1-43 endocytic process in fibroblasts isolated from wild-type mice, related to Fig. 7i. The time-lapse movie was obtained for 50 minutes with 30-second intervals using a confocal microscope (Leica SP8 TCS). The display rate is 7 frames per second.

**Supplementary Movie 2. FM1-43 endocytic process by fibroblasts from FAM92A1^+/-^ mice.**

Time-lapse movie of FM1-43 endocytic process in fibroblasts isolated from FAM92A1^+/-^ mice, related to Fig. 7i. The time-lapse movie was obtained for 50 minutes with 30-second intervals using a confocal microscope (Leica SP8 TCS). The display rate is 7 frames per second.

**Supplementary Movie 3. FM1-43 endocytic process by fibroblasts from FAM92A1^-/-^ mice.**

Time-lapse movie of FM1-43 endocytic process in fibroblasts isolated from FAM92A1^-/-^ mice, related to Fig. 7i. The time-lapse movie was obtained for 50 minutes with 30-second intervals using a confocal microscope (Leica SP8 TCS). The display rate is 7 frames per second.

**Supplementary Movie 4. The binding process of FAM92A1 monomer to PIP2 bilayer.**

The protein is shown in light blue. Positively charged amino acids are highlighted–lysine in orange and arginine in magenta. The phospholipid head groups are shown as teal spheres. PI(4,5)P_2_ molecules that initially make contact with FAM92A1 monomer have been highlighted. Oxygen atoms are shown as red spheres and carbon atoms are shown in cyan. Hydrogen atoms are not shown. The remaining PI(4,5)P_2_ molecules as well as all other membrane phospholipids, water, and ions are not shown for clarity. Time period shown: 0–300 ns.
