# Supplementary material for: Membrane remodeling by FAM92A1 during brain development regulates neuronal morphology, synaptic function, and cognition
Source: Nat Commun. 2024 Jul 23;15:6209. doi: 10.1038/s41467-024-50565-w (PMC11266426; doi:10.1038/s41467-024-50565-w)
Supplement: Supplementary file 8 — Reporting Summary [file 41467_2024_50565_MOESM8_ESM.pdf]

nature portfolio

Corresponding author(s): Xiaobo Cen

Last updated by author(s): Jul 6, 2024

Reporting Summary

Nature Portfolio wishes to improve the reproducibility of the work that we publish. This form provides structure for consistency and transparency in reporting. For further information on Nature Portfolio policies, see our Editorial Policies and the Editorial Policy Checklist.

Statistics

For all statistical analyses, confirm that the following items are present in the figure legend, table legend, main text, or Methods section.

n/a	Confirmed	
☐	☒	The exact sample size (*n*) for each experimental group/condition, given as a discrete number and unit of measurement
☐	☒	A statement on whether measurements were taken from distinct samples or whether the same sample was measured repeatedly
☐	☒	The statistical test(s) used AND whether they are one- or two-sided<br>*Only common tests should be described solely by name; describe more complex techniques in the Methods section.*
☐	☒	A description of all covariates tested
☐	☒	A description of any assumptions or corrections, such as tests of normality and adjustment for multiple comparisons
☐	☒	A full description of the statistical parameters including central tendency (e.g. means) or other basic estimates (e.g. regression coefficient) AND variation (e.g. standard deviation) or associated estimates of uncertainty (e.g. confidence intervals)
☐	☒	For null hypothesis testing, the test statistic (e.g. *F*, *t*, *r*) with confidence intervals, effect sizes, degrees of freedom and *P* value noted<br>*Give P values as exact values whenever suitable.*
☒	☐	For Bayesian analysis, information on the choice of priors and Markov chain Monte Carlo settings
☒	☐	For hierarchical and complex designs, identification of the appropriate level for tests and full reporting of outcomes
☒	☐	Estimates of effect sizes (e.g. Cohen's *d*, Pearson's *r*), indicating how they were calculated

Our web collection on statistics for biologists contains articles on many of the points above.

Software and code

Policy information about availability of computer code

Data collection	All software is commercially or freely available. Fluorescence images were captured using the N-STORM microscope (ECLIPSE Ti-E, Nikon) or a Lecia TCS SP8 laser scanning microscope. Electron micrographs were captured using transmission electron microscopy (FEI, Tacnai, G2 spirit, Hillsboro, Oregon), Zeiss FIB-SEM (Carl Zeiss microscopy BmbH, Jena, Germany), or a JEM-1400PLUS (JEOL ltd, Japan). Flow cytometry analysis was performed on a BD Fortessa X20 flow cytometer (BD Biosciences) using the BD FACS Diva™ software version 8.0 (BD Biosciences). For Golgi staining and IHC analysis, slides were photographed by a digital slide scanner (Pannoramic MIDI, 3DHISTECH Ltd., Hungary). All luminescence and absorbance assays on the plate were conducted using a multimode plate reader (Varioskan Flash, ThermoFisher). Quantitative PCR reactions were carried out in QuantStudio 1 Real-Time PCR System (QS-1) (ThermoFisher). Mitochondrial oxygen consumption rate (OCR) was measured using a Seahorse XFp Extracellular Flux Analyzer (Seahorse Bioscience, Billerica, MA). For RNA-seq, the Illumina NovaSeq 4000 was used. For MRI scans, all mice were scanned with a small animal 7T magnetic resonance imaging system (BioSpec 70/30USR, Bruker, Ettlingen, Germany) or a small animal 7T (MRINOVA 7.0T/16, Time Medical, China).The structure data were collected at Diamond Light source beamline I24. All-atom molecular dynamics simulations were performed using the GROMACS simulation package ver. 2022.5. The MEA data were recorded with the Maestro Edge multi-well MEA system (Axion Biosystems Inc., Atlanta, GA, USA). In whole-cell patch-clamp recording, all signals were acquired with a MultiClamp 700B amplifier (Molecular Devices), filtered at 1 kHz, and sampled at 5 kHz with a Digidata 1440A interface using Clampex 10.2 (Molecular Devices). The calcium signals were recorded with a LabVIEW program (Thinker Tech, Nanjing, China). Mouse behavior were recorded using the ANY-maze software (Stoelting CO., LTD, Wood Dale, IL, USA) and the EthoVision 7.0 software (EthoVision 7.0; Noldus Information Technology, Leesburg, VA, USA).
Data analysis	All software is commercially or freely available. Flow cytometry analysis was performed by FlowJo software (version 10.8.1). Spines were then automatically reconstructed and manually adjusted using the Filament Tracer module of Imaris (Bitplane). The structure of the FAM92A1 BAR dimer was processed with AutoPROC (https://www.globalphasing.com/autoproc/). The analysis of the simulated FAM92A1 BAR dimer was performed using standard GROMACS tools. Data of mitochondrial oxygen consumption rate were analyzed using the Seahorse Wave 2.6.3

software (Seahorse Bioscience). GSEA was performed using GSEA software (http://www.gsea-msigdb.org/gsea/) with 1000 permutations. Gene sets used were obtained from MSigDB (Hallmark gene sets; Reactome subset of the canonical pathway from C2 databases). The involved diseases of DEGs were enriched with Ingenuity Pathway Analysis software. The reconstruction of the mouse brain using the 7T MRI data was carried out with Mimics software. The raw 7T MRI data converted to NIFTI format using the MRIcroGL package (nitrc.org/projects/mricrogl/) and analyzed software (SPM12, Wellcome Trust Centre for Neuroimaging, London, UK) in MATLAB 2013b (MathWorks, Cambridge, United Kingdom). The quantification of synaptic vesicles was performed by MIB software. The whole-cell patch-clamp recording data were analyzed by MiniAnalysis 6.07 software (Synaptosoft). MEA data was analyzed by Neural Metric Tool (Axion Biosystems). The GCaMP6m fluorescence signals were analyzed in MATLAB 2016a software (MathWorks, Cambridge, United Kingdom). Statistical Analysis was done in GraphPad Prism 9, SPSS software (version 27.0, IBM Corporation). Figures were compiled using Fiji (v2.0.0-rc-69/1.52p)and CoreDRAW (version 24.0). Schematic diagrams in Fig.3e, l; 5a and Supplementary Fig. 1c; 4g, j; 5a are created CoreDRAW.

For manuscripts utilizing custom algorithms or software that are central to the research but not yet described in published literature, software must be made available to editors and reviewers. We strongly encourage code deposition in a community repository (e.g. GitHub). See the Nature Portfolio guidelines for submitting code & software for further information.

Data

Policy information about availability of data

All manuscripts must include a data availability statement. This statement should provide the following information, where applicable:

- Accession codes, unique identifiers, or web links for publicly available datasets
- A description of any restrictions on data availability
- For clinical datasets or third party data, please ensure that the statement adheres to our policy

The FIB-SEM data was uploaded and accessible on EMPIAR (ID: 12041) (https://doi.org/10.6019/EMPIAR-12041). The RNA-seq data have been deposited in NCBI's Gene Expression Omnibus (GEO) and are available through the GEO series accession number GSE264202. The crystal structure of FAM92A1 BAR domain protein structure coordinates has been deposited to PDB with the accession code 8CEG (PDB ID 8CEG) (https://www.rcsb.org/structure/8CEG).

Research involving human participants, their data, or biological material

Policy information about studies with human participants or human data. See also policy information about sex, gender (identity/presentation), and sexual orientation and race, ethnicity and racism.

Reporting on sex and gender	*Use the terms sex (biological attribute) and gender (shaped by social and cultural circumstances) carefully in order to avoid confusing both terms. Indicate if findings apply to only one sex or gender; describe whether sex and gender were considered in study design; whether sex and/or gender was determined based on self-reporting or assigned and methods used.* *Provide in the source data disaggregated sex and gender data, where this information has been collected, and if consent has been obtained for sharing of individual-level data; provide overall numbers in this Reporting Summary. Please state if this information has not been collected.* *Report sex- and gender-based analyses where performed, justify reasons for lack of sex- and gender-based analysis.*
Reporting on race, ethnicity, or other socially relevant groupings	*Please specify the socially constructed or socially relevant categorization variable(s) used in your manuscript and explain why they were used. Please note that such variables should not be used as proxies for other socially constructed/relevant variables (for example, race or ethnicity should not be used as a proxy for socioeconomic status).* *Provide clear definitions of the relevant terms used, how they were provided (by the participants/respondents, the researchers, or third parties), and the method(s) used to classify people into the different categories (e.g. self-report, census or administrative data, social media data, etc.)* *Please provide details about how you controlled for confounding variables in your analyses.*
Population characteristics	*Describe the covariate-relevant population characteristics of the human research participants (e.g. age, genotypic information, past and current diagnosis and treatment categories). If you filled out the behavioural & social sciences study design questions and have nothing to add here, write "See above."*
Recruitment	*Describe how participants were recruited. Outline any potential self-selection bias or other biases that may be present and how these are likely to impact results.*
Ethics oversight	*Identify the organization(s) that approved the study protocol.*

Note that full information on the approval of the study protocol must also be provided in the manuscript.

Field-specific reporting

Please select the one below that is the best fit for your research. If you are not sure, read the appropriate sections before making your selection.

☒ Life sciences ☐ Behavioural & social sciences ☐ Ecological, evolutionary & environmental sciences

For a reference copy of the document with all sections, see nature.com/documents/nr-reporting-summary-flat.pdf

Life sciences study design

All studies must disclose on these points even when the disclosure is negative.

Sample size	To meet the required sample size for conducting ANCOVA analysis of g-ratio in Fig.6h, we determined the necessary sample size using G*Power 3.1 prior to performing the statistical analysis.<br>For in vivo mouse experiments, we determined the sample size based on our extensive experience with animal models and endpoints, as well as the published articles on animal numbers for behavioral tests. Mice were randomly and blindly tested during the behavioral test.<br>For in vitro assays, a minimum of n = 3 biological or technical experiments were used for each analysis, following standard practices in the research field. This selection was made to produce reproducible results with a significance level of less than 0.05 and a power exceeding 90%.
Data exclusions	No data were excluded from analyses
Replication	Replicates were set up in all cell line experiments and animal experiments as showed in text, figure legends. All other experiments have been repeated at least twice with consonant results.
Randomization	Mice were randomized and blindly grouped and detected during the experimental procedures. All the cell experiments in vitro were performed by plating the cells in independent dishes or plates, and randomly detected without intrtroduced bias. Detailed definitions and descriptions were provided in the manuscript.
Blinding	The investigators were kept unaware of group allocation throughout data collection and analysis whenever feasible. Mice were detected and analyzed without genotype information by the experimenters. In vitro assays were conducted and imaged blindly by two operators. Data analysis involved at least two investigators to ensure the accuracy of quantification.

Reporting for specific materials, systems and methods

We require information from authors about some types of materials, experimental systems and methods used in many studies. Here, indicate whether each material, system or method listed is relevant to your study. If you are not sure if a list item applies to your research, read the appropriate section before selecting a response.

Materials & experimental systems

n/a	Involved in the study	
☐	☒	Antibodies
☐	☒	Eukaryotic cell lines
☒	☐	Palaeontology and archaeology
☐	☒	Animals and other organisms
☒	☐	Clinical data
☒	☐	Dual use research of concern
☒	☐	Plants

Methods

n/a	Involved in the study	
☐	☐	ChIP-seq
☐	☒	Flow cytometry
☐	☒	MRI-based neuroimaging

Antibodies

Antibodies used	Primary antibodies used for Western blot analysis: rabbit anti-FAM92A1 (1:500, Cat# HPA034760, Sigma), rabbit anti-FAM92A1 (1:1000, Cat# 24803-1-AP, Proteintech), rabbit anti-Dynamin 2 (1:4000, Cat# 14605-1-A, Proteintech), rabbit anti-CLCa (1:1000, Cat# 10852-1-AP, Proteintech), rabbit anti-SNX9 (1:8000, Cat# 15721-1-AP, Proteintech), rabbit anti-Synaptotagmin-1 (1:1000, Cat# 14511-1-AP, Proteintech), rabbit anti-vGlut1 (1:500, Cat# 55491-1-AP, Proteintech), mouse anti-PSD95 (1:2000, Cat# MA1-046, Invitrogen), rabbit anti-SDHA (1:1000; Cat# 14865-1-AP; Proteintech), rabbit anti-Synaptophysin (1:20,000, Cat# ab32127, Abcam), rabbit anti-GAPDH (1:2000, Cat# 2118, Cell Signaling Technology), rabbit anti-β-actin(1:50000, Cat# 81115-1-RR, Proteintech), rabbit anti-α-Tubulin(1:2000, Cat# 2125, Cell Signaling Technology).<br>Primary antibodies used for immunofluorescence, immunohistochemistry, and Immuno-EM are listed as follows: rabbit anti-FAM92A1 (1:100, Cat# HPA034760, Sigma), rabbit anti-FAM92A1 (1:100, Cat# 24803-1-AP, Proteintech), rabbit anti-TOM20 (1:1000, Cat# 11802-1-AP, Proteintech), mouse anti-VDAC (1:50, Cat# ab14734, Abcam), mouse anti-Clathrin (1:50, Cat# 610449, BD Bioscience), mouse anti-Caveolin (1:50, Cat# 610406, BD Bioscience), mouse anti-PSD95 (1:500, Cat# MA1-046, Invitrogen), mouse anti-SV2 (1:250, Cat# SV2, DSHB), mouse anti-MAP2 (1:400, Cat# 13-1500, Invitrogen), rabbit anti-vGlut1 (1:1000, Cat# 55491-1-AP, Proteintech), rabbit anti-Vimentin (1:500, Cat# 10366-1-AP, Proteintech). Actin was visualized with Alexa Fluor 647 conjugated to phalloidin (1:200; Cat# A22287, Invitrogen).
Validation	All antibodies used in this study are commercially available and were otherwise validated by the manufacturer, by previous studies from other laboratories, or by previous studies from our laboratory, as cited in the text and methods. For example, we used CRISPR-Cas9 to knockout the target proteins in mice and validated knockout by western blot using different mouse tissues.

Eukaryotic cell lines

Policy information about cell lines and Sex and Gender in Research

Cell line source(s)	The primary fibroblast cells were isolated from three FAM92A1 genotyping mice and were cultured in 1640 medium supplemented with 10% FBS, 100 U/mL penicillin, 100 µg/mL streptomycin, and 0.25 µg/mL amphotericin B. Cells were identified by immunofluorescence using the Vimentin antibody.<br>The primary hippocampal neurons were isolated wild-type embryos of C57 mice. The dissociated hippocampal cells were cultured with neurobasal medium supplemented with 2% B27 supplement (Gibco), 0.5 mg/mL primocin, and 0.5 mM glutamine. Neurons were maintained in a humidified 5% CO2 atmosphere at 37℃, and half of the medium was changed every three days. Neurons were identified immunofluorescence using the MAP2 antibody.<br>The mouse hippocampus-derived neuronal cell line HT22 cells were obtained from the Cell Bank of Chinese Academy of Science (Serial# GNM47, Shanghai, China). HT22 cells were cultured in DMEM medium (Cat# 11965092, Gibco) supplemented with 10% FBS, 1% penicillin-streptomycin-glutamine (100×) (Cat# 10378016, Gibco). Cells were incubated in a humidified 5% CO2 atmosphere at 37℃.
Authentication	The primary fibroblast cells and primary hippocampal neurons were authenticated by immunofluorescence in the lab using Vimentin and MAP2 antibodies, respectively. HT22 cells were authenticated by fingerprinting using short tandem repeat testing.
Mycoplasma contamination	All cell lines were verified to be free of mycoplasma contamination.
Commonly misidentified lines<br>(See ICLAC register)	No commonly misidentified cell lines were used in this study.

Animals and other research organisms

Policy information about studies involving animals; ARRIVE guidelines recommended for reporting animal research, and Sex and Gender in Research

Laboratory animals	For the generation of FAM92A1 knockout mice, the FAM92A1 heterozygotes (FAM92A1+/-) and homozygotes (FAM92A1-/-) were purchased from GemPharmatech (Nanjing, China).<br>For Alzheimer's disease model mice, eighteen-month-old male APPswe/PS1ΔE9 (abbreviated as APP/PS1 mice) transgenic mice and age matched wild-type mice were purchased from Beijing HFK Bioscience Co., Ltd. (Beijing, China; certification number SCXK 2014-0004).<br>For FAM92A1 knockdown experiments in vivo, adult male C57BL/6J mice (6–8 weeks old, 20–25 g) were purchased from GemPharmatech Co., Ltd. (Nanjing, China).<br>All mice were housed in cages with 4–5 mice per cage in a controlled environment with a 12-hour light/12-hour dark cycle (lights on at 7:00 a.m. and off at 7:00 p.m.), a constant ambient temperature of 23 ± 3°C, and humidity of 55 ± 5%.
Wild animals	No wild animals were used in the study.
Reporting on sex	Sex was not considered in the study design. Both male and female mice were used for breeding and experiments in this study.
Field-collected samples	No field-collected samples were used in this study.
Ethics oversight	The animal experiments were performed in strict accordance with the People's Republic of China legislation regarding the use and care of laboratory animals. All procedures used in this study were approved by the Institutional Animal Care and Use Committee of Sichuan University (permit number: 20240428001). All the efforts were made to minimize the suffering of the mice.

Note that full information on the approval of the study protocol must also be provided in the manuscript.

Plants

Seed stocks	*Report on the source of all seed stocks or other plant material used. If applicable, state the seed stock centre and catalogue number. If plant specimens were collected from the field, describe the collection location, date and sampling procedures.*
Novel plant genotypes	*Describe the methods by which all novel plant genotypes were produced. This includes those generated by transgenic approaches, gene editing, chemical/radiation-based mutagenesis and hybridization. For transgenic lines, describe the transformation method, the number of independent lines analyzed and the generation upon which experiments were performed. For gene-edited lines, describe the editor used, the endogenous sequence targeted for editing, the targeting guide RNA sequence (if applicable) and how the editor was applied.*
Authentication	*Describe any authentication procedures for each seed stock used or novel genotype generated. Describe any experiments used to assess the effect of a mutation and, where applicable, how potential secondary effects (e.g. second site T-DNA insertions, mosiacism, off-target gene editing) were examined.*

ChIP-seq

Data deposition

☐ Confirm that both raw and final processed data have been deposited in a public database such as GEO.

☐ Confirm that you have deposited or provided access to graph files (e.g. BED files) for the called peaks.

Data access links<br>*May remain private before publication.*	*For "Initial submission" or "Revised version" documents, provide reviewer access links. For your "Final submission" document, provide a link to the deposited data.*
Files in database submission	*Provide a list of all files available in the database submission.*
Genome browser session<br>(e.g. UCSC)	*Provide a link to an anonymized genome browser session for "Initial submission" and "Revised version" documents only, to enable peer review. Write "no longer applicable" for "Final submission" documents.*

Methodology

Replicates	*Describe the experimental replicates, specifying number, type and replicate agreement.*
Sequencing depth	*Describe the sequencing depth for each experiment, providing the total number of reads, uniquely mapped reads, length of reads and whether they were paired- or single-end.*
Antibodies	*Describe the antibodies used for the ChIP-seq experiments; as applicable, provide supplier name, catalog number, clone name, and lot number.*
Peak calling parameters	*Specify the command line program and parameters used for read mapping and peak calling, including the ChIP, control and index files used.*
Data quality	*Describe the methods used to ensure data quality in full detail, including how many peaks are at FDR 5% and above 5-fold enrichment.*
Software	*Describe the software used to collect and analyze the ChIP-seq data. For custom code that has been deposited into a community repository, provide accession details.*

Flow Cytometry

Plots

Confirm that:

☒ The axis labels state the marker and fluorochrome used (e.g. CD4-FITC).

☒ The axis scales are clearly visible. Include numbers along axes only for bottom left plot of group (a 'group' is an analysis of identical markers).

☒ All plots are contour plots with outliers or pseudocolor plots.

☒ A numerical value for number of cells or percentage (with statistics) is provided.

Methodology

Sample preparation	Cells was trypsinized and harvested to create cell suspensions in PBS.
Instrument	BD Fortessa X20 flow cytometer (BD Biosciences)
Software	BD FACS Diva™ software version 8.0
Cell population abundance	Cell population data were collected on a debris exclusion gate at the time of acquisition of the BD Fortessa X20 flow cytometer. At least 10,000 cell events per sample were collected for each sample.
Gating strategy	Cell populations were gated on FSC/SSC for cell selection and debris exclusion. Next, a FSC-H/FSC-A plot was used to exclude the doublets. Live cells were further quantified by FITC to determine positive cell populations.

☒ Tick this box to confirm that a figure exemplifying the gating strategy is provided in the Supplementary Information.

Magnetic resonance imaging

Experimental design

Design type	Investigate the reason for brain swelling after loss of FAM92A1.
Design specifications	For T2-weighted MRI, the sequence was referred from the published articles with minor modification after based on

the quality of MR images. The time of scanning were spent around 30 min for single mouse. For T2-weighted 3D fast spin echo (FSE) sequence, the sequence were also referred from the previous reported sequence with modification based on the quality of images and time consuming. The time of scanning were spent around 4 h for single mice.

Behavioral performance measures

For T2-weighted MRI, at least eight mice per group were used for MRI scanns. For T2-weighted 3D fast spin echo (FSE) sequence, at least three mice were used for each group. The number of animals used for T2-weighted MRI is mainly determined based on the published articles. For T2-weighted 3D fast spin echo sequence, due to time consuming nature, at least 4 hours were consumed for singled mice, hence, three mice for each group were used for scanning and final analysis. No data were excluded.

Acquisition

Imaging type(s)

T2-weighted MRI (T2W-MRI) and T2-weighted 3D fast spin echo (FSE) sequence

Field strength

7T magnetic resonance imaging system

Sequence & imaging parameters

A high-resolution rapid acquisition with relaxation enhancement (Turbo-RARE) scan was used for anatomical, T2-weighted MRI (T2W-MRI) with the following parameters as previously reported 84: matrix size, 256 × 256; slices, 28; slice thickness, 0.4 mm (no gap); field of view (FOV), 17.5 × 17.5 mm2; repetition time (TR), 5500 ms; echo time, 32.5 ms; echo spacing, 10.833 ms; rare factor, 8; flip angle, 90; refocusing angle, 180.

MRI scans of perfused mice were performed on a small animal 7T (MRINOVA 7.0T/16, Time Medical, China). Each perfused brain with skull was carefully fixed on the animal carrier. A referred T2-weighted 3D fast spin echo (FSE) sequence was used with minor modification 82: TR= 2000 ms; ESP = 42 ms; ETL = 8; effective TE = 84 ms; number of averages = 1; FOV = 25 mm × 20 mm × 20 mm; and matrix size = 384 × 192 × 192.

Area of acquisition

To adjust the mouse position and perform a two-step shim procedure (first global shim, followed by local shim on the brain volume), initial sequences (Localizer, Localizer multislice, and Calcshim) were acquired.

Diffusion MRI ☐ Used ☒ Not used

Preprocessing

Preprocessing software

For VBM analysis, The PCNN3D was used to segment non-brain tissue for in vivo scans; The SPM12 toolbox was used to extract whole brain tissues were then extracted; ITK-SNAP was applied to decorate a flawed mask. For DBM analysis, the dcm2niix tool included in the MRIcroGL package was used to convert the raw diffusion data were first converted to NIFTI format. For ventricle area, the area of each ventricle was measured using the Fiji software.

Normalization

All MRI scans of individual subjects were orderly linearly and non-linearly registered using ANTS

Normalization template

Template mouse brain from Allen institute

Noise and artifact removal

The Turone Mouse Brain Atlas and Template (TMBT) was applied to nonlinearly normalize the tissue class images.

Volume censoring

The local Jacobian determinants were then applied to characterize volume difference at each voxel (3D pixel) between two groups.

Statistical modeling & inference

Model type and settings

Comparisons between two groups were conducted using a student's t-test (two-tailed) using the statistical analyzer in SPM12. Voxel $p < 0.05$, cluster $p < 0.05$ and family-wise error (FWE) < 0.05 were set as the cut-off for statistical significance. Contrast T-maps were compared voxel by voxel with one-way analysis of variance F-test with a gray matter mask settled at an absolute threshold mask of 0.2 (with relative total brain volumes entered as a covariate). For multiple comparisons, the stringent familywise error correction was applied with a statistical threshold of 0.05.

Effect(s) tested

one-way analysis of variance F-test with a gray matter mask settled at an absolute threshold mask of 0.2. Voxel $p < 0.05$, cluster $p < 0.05$ and family-wise error (FWE) < 0.05 were set as the cut-off for statistical significance.

Specify type of analysis: ☒ Whole brain ☐ ROI-based ☐ Both

Statistic type for inference

(See Eklund et al. 2016)

The stringent familywise error correction was applied with a statistical threshold of 0.05 and a cluster size of 200 voxels.

Correction

Voxel $p < 0.05$, cluster $p < 0.05$ and family-wise error (FWE) < 0.05

Models & analysis

n/a	Involved in the study
☒	☐ Functional and/or effective connectivity
☐	☒ Graph analysis
☒	☐ Multivariate modeling or predictive analysis

Graph analysis

Ventricular dilatation, along with alterations in the volumetric morphology of brain areas, and decreased gray matter volume around the multiple brain areas (area 1, encompassing multilayers of entorhinal area, medial part, dorsal zone-ENTm; area 2, encompassing multilayers of the retrosplenial area-RSP, corpus callosum, and cingulum bundle), may contribute to brain swelling in FAM92A1-depleted mice.
